# Genetic and Physiological Characterization of a Calcium Deficiency Phenotype in Maize

**DOI:** 10.1534/g3.120.401069

**Published:** 2020-04-01

**Authors:** Yanli Wang, Lais Bastos Martins, Shannon Sermons, Peter Balint-Kurti

**Affiliations:** * Dept. of Entomology and Plant Pathology, NC State University, Raleigh NC 27695; † Key Laboratory of Biology and Genetic Improvement of Maize in Southwest Region, Maize Research Institute, Sichuan Agricultural University, Chengdu 611130, China; ‡ Plant Science Research Unit, USDA-ARS, NC State University, Raleigh NC, 27695

**Keywords:** QTL, Calcium deficiency, maize

## Abstract

Calcium (Ca) is an essential plant nutrient, required for signaling, cell wall fortification and growth and development. Calcium deficiency (Ca-deficiency) in maize causes leaf tip rot and a so-called “bull-whipping” or “buggy-whipping” phenotype. Seedlings of the maize line B73 displayed these Ca-deficiency-like symptoms when grown in the greenhouse with excess fertilizer during the winter months, while seedlings of the Mo17 maize line did not display these symptoms under the same conditions. These differential phenotypes could be recapitulated in ‘mini-hydroponic’ systems in the laboratory in which high ammonium, but not nitrate, levels induced the symptoms in B73 but not Mo17 seedlings. Consistent with this phenotype being caused by Ca-deficiency, addition of Ca^2+^ completely relieved the symptoms. These data suggest that ammonium reduces the seedling’s ability to absorb calcium, which causes the Ca-deficiency phenotype, and that this trait varies among genotypes. A recombinant inbred line (RIL) population derived from a B73 x Mo17 cross was used to map quantitative trait loci (QTL) associated with the Ca-deficiency phenotype. QTL associated with variation in susceptibility to Ca-deficiency were detected on chromosomes 1, 2, 3, 6 which explained between 3.30–9.94% of the observed variation. Several genes predicted to bind or be activated by calcium map to these QTL on chromosome 1, 2, 6. These results describe for the first time the genetics of Ca-deficiency symptoms in maize and in plants in general.

Calcium (Ca) is an essential plant nutrient playing multiple roles in the cell. It is important for membrane stability, cell integrity, cell division and elongation (Steward 1974; Kirkby and Pilbeam 1984; White and Broadley 2003) and for multiple signal transduction pathways in which fluctuations in Ca^2+^ concentrations are important for activation (Monshausen 2012). Maize is crucial to global food security because of its widespread use in food, biofuels, and feed (Shiferaw *et al.* 2011). While Ca-deficiency symptoms are not often observed in field grown maize, they can sometimes be observed in sandy soil or in soils which have been over-fertilized (Melsted 1953).

The majority of Ca uptake in plants is believed to be largely passive via the xylem and apoplast, meaning that transpiration and water movement through plants is crucial for Ca uptake (White and Broadley 2003; Gilliham *et al.* 2011). Ca does not have the ability to move from the older to young tissue via the phloem. This causes developing tissue to rely on the water movement through the xylem for its supply of Ca. The transport of ions in the xylem depends on transpiration, so plants may show a Ca-deficiency phenotype under low transpiration (White and Broadley 2003). Ca-deficiency symptoms often occur not because of lack of Ca in the soil but rather because of inhibition of Ca^2+^ uptake due to competition for binding sites with other cations such as Na^+^, K^+^, Mg^2+^ and NH_4_^+^ (Maas and Grieve 1987). Ca^2+^ uptake in maize has been reported to be inhibited by excess NH_4_^+^ application in both field and greenhouse conditions (Melsted 1953; Wilcox *et al.* 1973; Kawaski and Moritsugu 1979).

In general, Ca-deficiency symptoms in most plants manifest as rot or necrosis at the extremities, the leaf tips or fruits. These symptoms are thought be due to the important role Ca plays in membrane integrity and in cell wall strengthening (Simon 1978). Ca-deficiency is not often observed in modern agriculture due to the relative abundance of Ca in most soils and regular liming with chemicals such as CaCO_3_ and Ca(OH)_2_. However, in untreated sandy soils or in intensive agriculture, where excessive fertilization leads to the buildup of other cations, Ca-deficiency can be a problem (Melsted 1953; Kirkby and Pilbeam 1984).

Ca-deficiency in maize can be identified by some specific symptoms. Typically, the young leaves become serrated, curl and rot. The outer leaf may curl over the next emerging leaf and prevent its expansion, leading to symptoms have been described as ‘bull-whip’ or ‘buggy-whip’ since the plant resembles a curled-up whip. Ca-deficiency was found to be a major factor limiting maize growth in in acidic, unlimed ultisols soils of eastern Nigeria, and, in these cases, treatment with CaSO_4_ significantly increased maize dry matter yield relative to the untreated soil (Njoku *et al.* 1987). Ca-deficiency symptoms are also quite commonly seen in the greenhouse under experimental conditions, especially during the winter months when transpiration is limited.

Variability among maize lines with respect to Ca uptake and deficiency symptoms has been noted previously. Considerable variations in Ca concentrations and a positive correlation (r = 0.49*, *P* < 0.01) between the Ca concentration of ear-leaf at flowering stage and grain yield was reported among nine maize lines (Kovačević *et al.* 2017). Clark and Brown (1974) reported some variation in Ca-deficiency symptoms among a set of maize inbreds.

This study was inspired by the observation that two maize lines commonly used in genetics, B73 and Mo17, differed for their Ca-deficiency symptoms. While typical bull-whip Ca-deficiency symptoms were often observed when B73 was grown in fertilized soil during the fall and winter months, no such symptoms were observed for Mo17. This afforded the opportunity to investigate the genetic basis of variation in Ca-deficiency symptoms in maize. The goals of this study were: (1) To confirm that the basis of the bull-whip symptoms observed in B73 were due to Ca-deficiency; (2) to characterize the genetic basis of variation in Ca-deficiency between B73 and Mo17.

## Material and Methods

### Plant Materials

In this study we used the maize lines B73, Mo17 and 276 inbred lines comprising the Intermated B73 x Mo17 (IBM) mapping population (Lee *et al.* 2002) (Table S1).

### Growth conditions and scoring of Ca-deficiency phenotype

Greenhouse experiments were conducted from November 2018 to March 2019 at the Method Rd. greenhouse complex in Raleigh NC. Greenhouse temperatures were set at 26° during the day and 22° at night. For every greenhouse experiment, five seeds were sown at ∼1 inch depth in each pot (diameter 15 cm, volume 1.5 L) in Fafard 4P Professional Growing Mix (Sun Gro Horticulture, Agawam, MA). When added, 13.3g Osmocote 19-6-12 or 14-14-14 (ICL Specialty Fertilizers, Summerville, SC) fertilizer per pot was mixed thoroughly with the soil before planting. This rate of 20 g L^-1^, about double the maximum recommended by the product label, was chosen to induce the phenotype based on a preliminary experiment conducted in May 2018. Experiments were arranged in a randomized block design.

The IBM population was screened twice, from November 26 to December 10 and from January 7 to January 14. The first replicate included one pot per inbred line with 13.3 g Osmocote (treatment) and one without (control), while the second replicate consisted of one pot of the 13.3 g Osmocote treatment per inbred line. The proportion of plants in each pot showing Ca-deficiency phenotype (bull-whipping) was recorded every 2 days after seedling emergence for 14 days for replication 1 and for 7 days for replication 2.

### Ionomic analysis

B73 plants were grown under the same greenhouse treatments described above during the fall and winter months. Three leaves from each of 20 B73 plants (the first, second and third youngest leaves) under control (only soil) and treatment (soil + Osmocote) conditions were collected at 13 days after planting, when the Ca-deficiency phenotype first appeared in the treatment conditions. Samples were dried in an oven at 37°. 250 mg dry tissue of each sample was sent to the Donald Danforth Plant Science Center ionomics facility for analysis by inductively coupled plasma mass spectrometry. This experiment was performed twice.

### Laboratory Hydroponic system

Seeds of B73 and Mo17 inbred lines were washed in 1% H_2_O_2_ for 5min, then rinsed with sterile water three times. The seeds were then germinated in rolled moist tissue paper for four days. Germinated seedlings were suspended in sponge plugs at the top of a 15ml tube with the radicle oriented downward and immersed in treatment solution. The treatment solutions consisted of varying concentrations of Osmocote, ammonium nitrate, or ammonium nitrate + calcium nitrate as described in the results. Osmocote fertilizer prills were pulverized prior to solution preparation to allow full solubility. Tubes were placed into a Percival E36L2 reach-in growth chamber (Percival Scientific, Perry, IA) with 16/8 hr light/dark period and constant 26° temperature. The experiment was conducted twice, with five seedlings per treatment in each repetition. Seedlings were scored for the Ca-deficiency phenotype after three and six days of exposure to the solution.

### Data analysis and QTL mapping

For individual environments, weighted average (WA) values were calculated for each replication in each environment. To do this, the average value of two consecutive ratings (the proportion of plants showing the Ca-deficiency phenotype) was obtained and multiplied by the number of days between the ratings. Values were then summed over all intervals, and then divided by the number of days of evaluation to determine the weighted average. WA is conceptually identical to standardized area under disease progress curve (sAUDPC) (Shaner and Finney 1977; Campbell and Madden 1990) but the term WA is used here to avoid confusion as no disease was present in this study.

Phenotypic descriptive analysis (mean, range, standard deviation, the coefficient of variation) was performed using the Descriptive Statistics model and Analysis of variance (ANOVA) of phenotype was performed using the General Linear Model using SPSS 20.0 software (SPSS, Chicago, IL, USA). Correlations were calculated using the “corrplot” package in R. The H^2^ (broad-sense heritability) was computed as described, H^2^ = ${\text{σ}}_{g}^{2}$/ (${\text{σ}}_{g}^{2}+{\text{σ}}_{gy}^{2}/r+{\text{σ}}_{e}^{2}/yr$), where ${\text{σ}}_{g}^{2}$, ${\text{σ}}_{gy}^{2}$, ${\text{σ}}_{e}^{2}$ are the genetic, genotype × replication interaction variance component, and residual error variance components, respectively; r is the number of replications; and y represents the number of environments (Knapp *et al.* 2010). Line least-square means (LSMEAN) value and their 95% confidence interval were estimated for each inbred line. And the LSMEAN value was calculated by the “lsmeans” package in R.

The WA scores for replications 1 and 2 (WA1 and WA2 respectively) and the LSMEAN value of the 276 inbred lines of the IBM recombinant inbred line (RIL) population were used to perform QTL mapping. 1339 SSR markers with an average distance of 4.67 cM between markers were used in this study (https://maizegdb.org/data_center/qtl-data). The QTL analysis was performed using ICIMapping software based on composite interval mapping (CIM) model. The parameters used were: walk speed 1.0 cM, 1000 permutations was used to determine the likelihood of odds ratio (LOD) threshold value at a significance level of 0.05 (Terekhov *et al.* 1997). Based on these permutations, a LOD score of 3.0 was used as a minimum to determine the presence of a QTL.

### Data availability

All phenotypic and genotypic data used in this study are included in Supplementary Files (or are publicly available as described. Further information and all data used in this study is additionally available from the corresponding author if required. Supplemental material available at figshare: https://doi.org/10.25387/g3.11594142.

## Results

### Phenotypic variation for Ca-deficiency symptoms among maize lines

**Figure 1:**
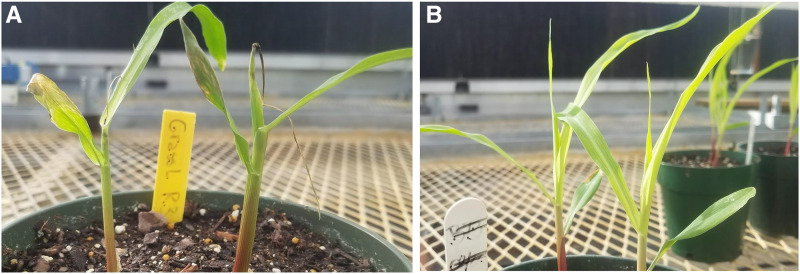
B73 seedlings grown with (A) and without (B) additions of 13.3g/pot Osmocote. These photos were taken 20 days after emergence in greenhouse.

In initial experiments conducted in the winter of 2017 two parents of the IBM population, B73 and Mo17, differed starkly in their expression of the leaf-rot/wilting, “bull-whipping” phenotype when grown in fall and winter with a high rate of Osmocote fertilizer. This bull-whip phenotype was described previously as a Ca-deficiency phenotype caused by excess fertilizer application (Kawaski and Moritsugu 1979) and we will subsequently refer to it here as the Ca-deficiency phenotype. All B73 plants showed the Ca-deficiency phenotype after 6 to 9 days of growth under such conditions, while none of the Mo17 seedlings showed symptoms. When unamended with fertilizer, neither B73 nor Mo17 showed the Ca-deficiency phenotype (Figure 1). These results were consistent across more than 50 plants grown under each condition. B73 plants tended to recover from the Ca-deficiency phenotype after 15 days of culture, but the leaf tips still had curling characteristics.

### Confirmation of Ca-deficiency phenotype

In order to confirm that the bull-whip phenotype we were observing was indeed due to Ca-deficiency, we took two approaches. First, we conducted elemental analysis to see if the level of Ca was indeed reduced in B73 seedlings grown under the fertilized conditions that induced the Ca-deficiency phenotype, compared to unfertilized conditions. Second, we developed a laboratory assay that allowed us to determine if we could rescue the phenotype by providing additional Ca.

#### Elemental analysis:

We determined the levels of elements in the leaves of B73 seedlings grown in control (unfertilized) and treatment (13.3g Osmocote fertilizer per pot) conditions. Leaves were harvested just as the Ca-deficiency phenotype started to appear at 13 days after planting. We performed two repetitions of this experiment. The Ca content in the seedling leaves was significantly higher under the control condition than under the treatment condition in the first repetition, but the trend was opposite in the second repetition (Table S2). Some heavy metals, such as Mn, Co were significantly lower in the control condition than in the treatment condition in both repetitions (Table S2). Elemental levels were largely in line with those reported in a previous study (Zdunić *et al.* 2014).

#### Phenotype rescue in hydroponic system:

**Figure 2:**
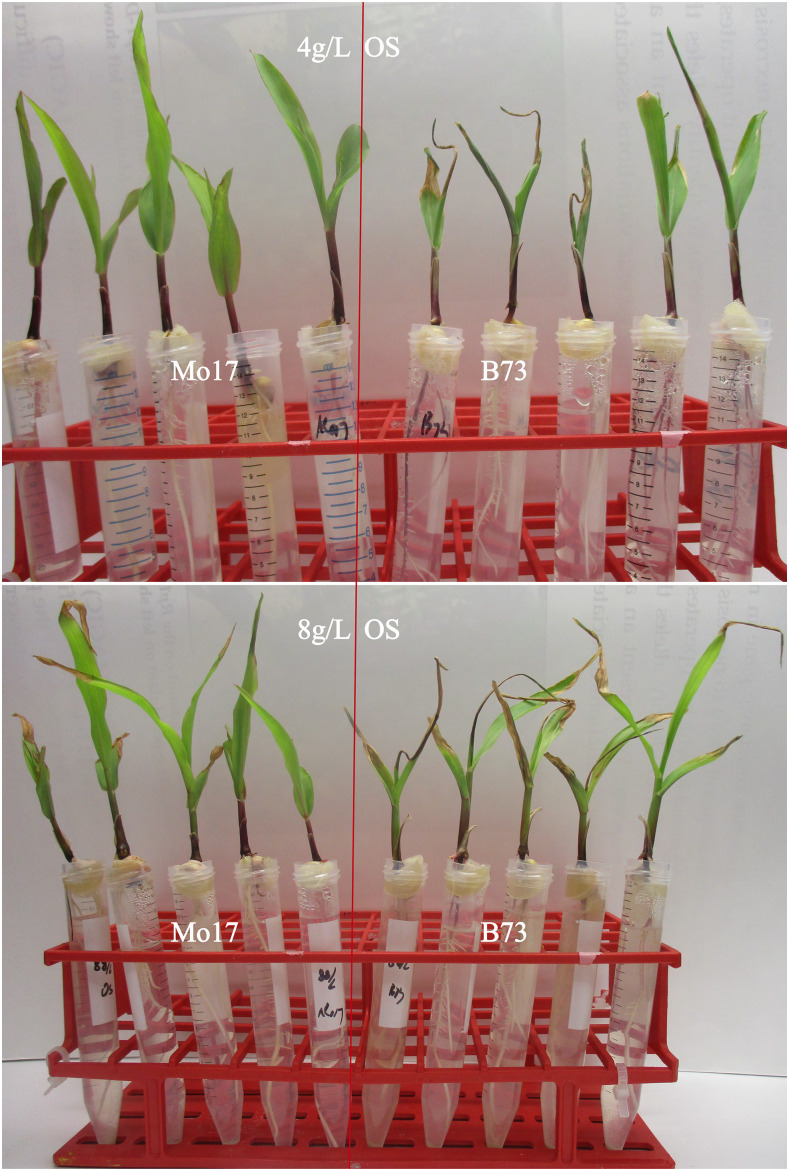
The Ca-deficiency phenotypes induced in lines B73 (right side) and Mo17 (left side) by 4g/L (top image) and 8g/L (bottom) Osmocote (OS) in the hydroponic laboratory assay. Osmocote was crushed and dissolved in dH_2_O.

We developed a ‘hydroponic’ system in the laboratory where germinating seedlings were suspended at the top of 15ml tubes containing different solutions that allowed us to precisely manipulate the concentration of ions to which the germinating seeds were exposed (Table S3). In this system, when grown in solution containing 4g/L and 8g/L dissolved Osmocote for six days, the youngest leaves of B73 seedlings were necrotic and the plants displayed the bull-whip phenotype, however, Mo17 just had minor yellow lesions at the tip of the leaf (Figure 2). No symptoms were apparent when either line was grown in water alone.

**Figure 3:**
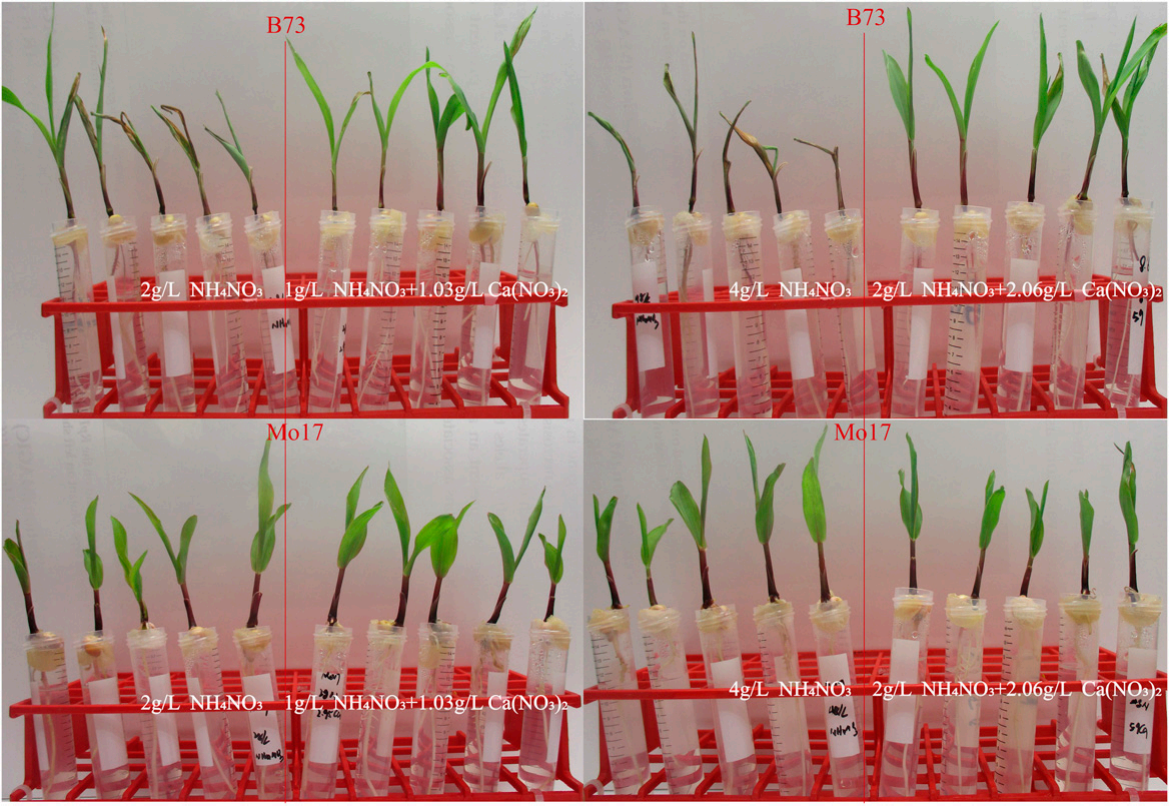
The Ca-deficiency phenotypes induced in lines B73 (top images as indicated) and Mo17 (bottom images) by different concentrations of NH_4_NO_3_ and Ca(NO3)_2_ as indicated. In each set of eight seedlings the four seedlings on the left were grown in NH_4_NO_3_ solution and the four on the right were grown in half the concentration of NH_4_NO_3_ supplemented with Ca(NO_3_)_2_ at a concentration adjusted to keep the concentration of NO_3_^-^ ions constant which meant that the NH_4_^+^/Ca^2+^ ratio was 2:1 in every case (see Table S3).

**Figure 4:**
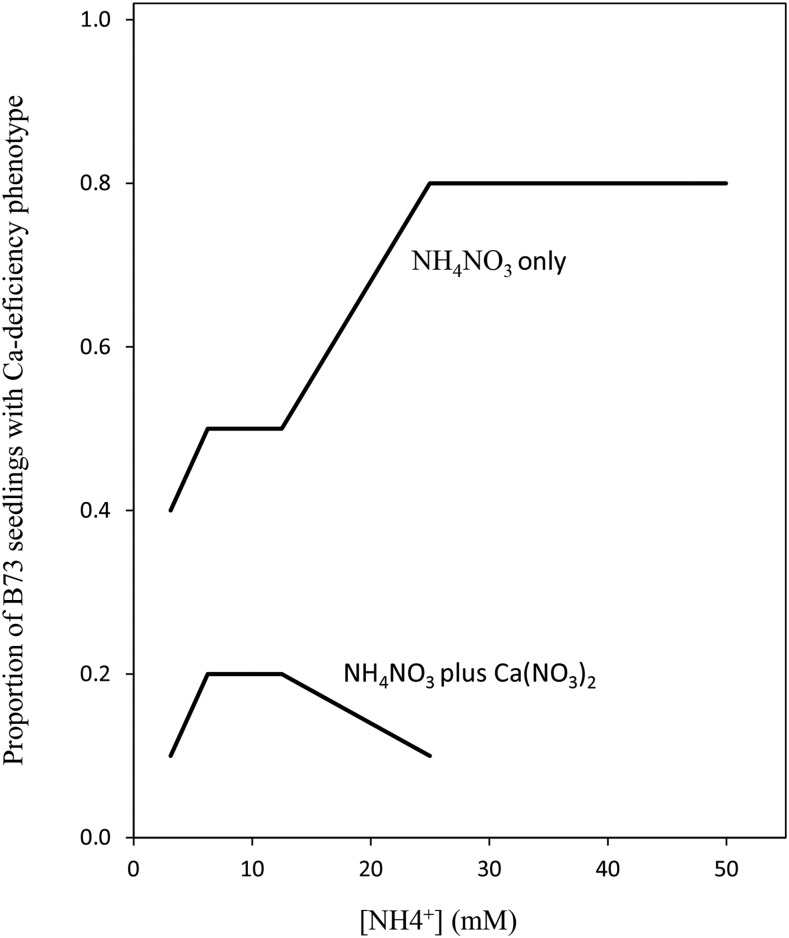
Proportion of B73 seedlings presenting the Ca-deficiency phenotype in two sets of treatments of the hydroponics assay: the NH_4_NO_3_ only and NH_4_NO_3_ plus Ca(NO3)_2_. Treatments are represented along the x axis in terms of NH_4_^+^ concentration. For the NH_4_NO_3_ plus Ca(NO3)_2_ treatments the amount of Ca(NO3)_2_ added provided the same concentration of NO_3_^-^ ions as the NH_4_NO_3_ which meant that the NH_4_^+^/Ca^2+^ ratio was 2:1 in every case (see Table S3).

Osmocote is a complex fertilizer consisting of NH_4_NO_3,_ (NH_4_)_3_PO_4_, K_2_SO_4_ and CaSO_4_, all of which are polymer-coated to ensure slow release. NH_4_NO_3_ is the majority constituent, 51.5% by weight, and NH_4_^+^ is therefore the major cation and is the most likely cause of inhibition of uptake of Ca^2+^ ions. To test whether NH_4_NO_3_ alone could cause the Ca-deficiency we grew B73 and Mo17 seedlings in 5 different concentrations of NH_4_NO_3_ from 0.25g/L to 4g/L in our hydroponic system (Table S3). After six days of culture, B73 seedling showed a much more apparent Ca-deficiency phenotype than Mo17 under all NH_4_NO_3_ concentrations (Figure 3, Table S3). We also showed that addition of Ca(NO_3_)_2_ rescued this phenotype (Figure 3, 4). These results strongly suggest that the bull-whip phenotype is caused by excess NH_4_^+^ and is due to reduction Ca^2+^ uptake caused by NH_4_^+^.

### QTL analysis of the Ca-deficiency phenotype

Since B73 and Mo17 showed contrasting phenotypes when grown in the fertilizer-amended soil, we used the IBM mapping population which was derived from a B73 x Mo17 cross (Lee *et al.* 2002) to map loci associated with variation in the appearance of the phenotype.

Ca-deficiency phenotype data and WA calculations for 276 inbred lines of the IBM population are presented in Table S4. Ca-deficiency symptoms were rarely seen in pots not amended with fertilizer, so all results reported below are for fertilizer-amended pots.

**Figure 5:**
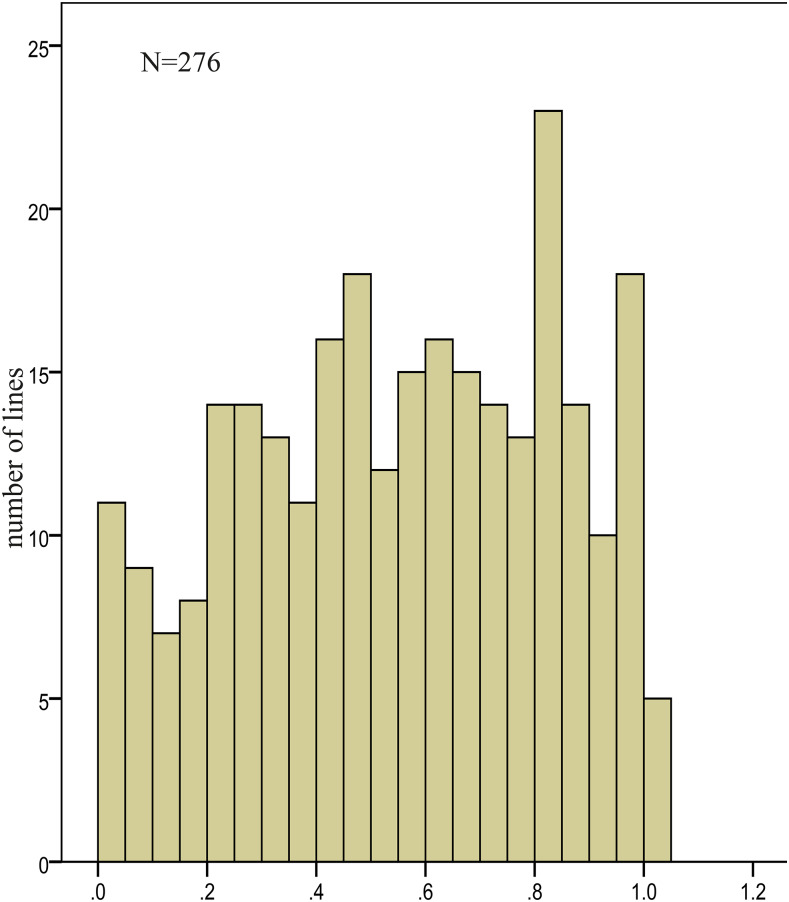
Frequency distribution of Ca-deficiency phenotype among 276 IBM RIL lines. X axis shows the weighted average (WA) of proportion of plants displaying the Ca-deficiency phenotype.

The 276 IBM RIL lines showed a wide range of variation for Ca-deficiency (Figure 5). The proportion of plants for each line displaying the Ca-deficiency phenotype were significantly correlated across the two replications (Fig. S1). The Pearson correlation coefficient of the weighted average (WA) of replications 1 and 2 was 0.66 (*P* < 0.001). The broad-sense heritability (H^2^) was high (0.73) indicating that the phenotype was strongly affected by genotype. This was borne out by the analysis of variance (Table 1).

**Table 1:** Analysis variation of WA of Ca-deficiency phenotype

Source of variation	Df	Sum Square	Mean Square	Sig.
Genotype(G)	275	41.816	0.152	<0.0001*^a^*
Replication	1	0.283	0.283	<0.0001*^a^*
G × R	254	8.461	0.033	<0.0001*^a^*

a indicate significance level at 0.01.

QTL associated with variation in the Ca-deficiency phenotype were calculated based on the WA1, WA2, and LSMEAN values. Five QTL were detected based on LSMEAN values, one on each of chromosomes 1, 2, 3 and two on chromosome 6, explaining between 3.32–9.94% of the phenotypic variation (Table 2, Fig. S2-5). Of these, the two strongest QTL at 376 cM on chromosome 2 and 293cM on chromosome 6 were detected in each replication and in the overall LSMEAN. Of the 5 QTL detected, B73 contributed the allele for the appearance of the Ca-deficiency phenotype for all except the QTL on chromosome 1. We examined the genes encoded at the QTL on chromosome 1, 2 and 6 of these QTL. At these QTL we identified 6 candidate genes in the B73 genome, including several with predicted functions which involve Ca (Table S5). No interactions between QTL were detected.

**Table 2:** QTL associated variation in Ca-deficiency phenotype in the IBM RIL population and associated parameter

Trait*^a^*	Chr*^b^*	Pos(cM)*^c^*	Support interval(cM)*^d^*	Physical position(bp)*^e^*	Physical interval (bp)*^f^*	Additive effect*^g^*	LOD*^h^*	R^2^(%)*^i^*
Rep1	1	233	229-235.5	41,790,546	40,174,500-42,688,350	0.0839	5.6746	6.7603
LSMEAN	1	234	230-235.5	42,149,668	40,357,405-42,688,350	0.0647	4.1878	4.6379
Rep1	2	374	371-378.5	195,054,537	193,854,729-196,597,148	−0.0811	5.0368	6.5182
Rep2	2	379	373-387.5	196,787,249	194,315,938-200,018,963	−0.0825	4.1921	5.8322
LSMEAN	2	376	372-379.5	195,646,644	193,935,736-196,977,350	−0.0936	7.58	9.9408
Rep1	3	22	15.5-28.5	1,706,793	1,509,963-2,016,301	−0.0588	3.0848	3.3016
LSMEAN	3	38	37-38.5	2,699,570	2,668,558-2,702,866	−0.0583	3.5284	3.8738
Rep2	6	89	87-92.5	78,511,697	76,390,730-81,481,050	−0.0736	3.5364	4.8418
LSMEAN	6	89	87-91.5	78,511,697	76,390,730-80,632,663	−0.0534	3.0025	3.3286
Rep1	6	296	289-301.5	151,281,214	150,328,028-151,980,218	−0.0958	4.8283	8.5598
Rep2	6	293	287-301.5	150,899,940	150,073,844-151,980,218	−0.0922	3.7728	6.7864
LSMEAN	6	293	287-300.5	150,899,940	150,073,844-151,853,127	−0.0889	5.628	8.3497

a QTL were calculated based on the trait values from replication 1 (Rep1), replication 2 (Rep2) and the overall LSMEAN values.

b Chromosome.

c Genetic position of QTL peak. It should be noted that since F2 plants were intermated 4 times before the production of the IBM RILS, the genetic map is expanded and centiMorgan (cM) values for the IBM population are not, strictly speaking, actually cM values indicating the average percentage of chromosomal crossovers in a single generation (Lee *et al.* 2002; Balint-Kurti *et al.* 2007)

d The genetic positions defining the 2 LOD support interval.

e The physical position of the QTL peak based on the B73 V4 genomic sequence.

f The physical positions defining the 2 LOD support interval.

g Additive effect: positive value indicates alleles for increased Ca-deficiency response contributed by Mo17, whereas negative value indicated alleles for increased Ca-deficiency response contributed by B73.

h The Log of Odds (LOD) value at each QTL.

i R^2^(%): percentage of phenotypic variance explained by QTL with additive effect; LOD threshold values for Osmocote fertilizer responses are 3.5.

## Discussion

Ca-deficiency symptoms have been observed in many species, causing disorders such as the blossom end rot in pepper, tomato and eggplants, tip burn in lettuce and bitter pit in apples (White and Broadley 2003). In all these cases, overfertilization is reported to be one of possible causes of these disorders. In sorghum and maize, symptoms such as curly and serrated leaf edges, and the appearance of the so-called “bull-whip” phenotype have been recognized as symptoms of Ca-deficiency (Melsted 1953; Kawaski and Moritsugu 1979; Murtadha *et al.* 2010).

In this study we observed a phenotype in maize seedlings grown with fertilizer amendment in the greenhouse that appeared to be consistent with Ca-deficiency symptoms (Figure 1) and to show genotype specificity. We demonstrated that the phenotype was indeed due to Ca-deficiency by rescuing the phenotype by adding Ca^2+^ in a laboratory assay (Figure 3, 4). We performed elemental analysis twice and found that the results were inconsistent both with respect to absolute concentrations of various elemental and with respect to relative levels between control and experimental (Osmocote) treatement. In these experiments, the three youngest leaves were taken from each inbred line shortly before the expression of Ca-deficiency symptoms, and the average calcium content was calculated. However the Ca-deficiency was generally expressed in the youngest leaf only. It is possible that the calcium content of the older two leaves was not reflective of that of the youngest and this may explain some of the inconsistencies we observed.

### Ca-deficiency and transpiration

Ca-deficiency symptoms in agricultural systems are rarely caused by lack of Ca in the soil but rather by deficiencies in the uptake into and transport of Ca through the plant (Bangerth 1979; Kirkby and Pilbeam 1984; White and Broadley 2003). Ca uptake is largely via apoplastic routes, particularly by transport through the xylem. Transport and accumulation of Ca is therefore significantly associated with transpiration rates (Gilliham *et al.* 2011). Since Ca, once deposited in cells is rarely redistributed (Hanger 1979), Ca-deficiency symptoms usually manifest in the growing extremities of the plant. Low transpiration therefore can lead to more serious Ca-deficiency symptoms (Clarkson 1984). In this study, experiments were performed in the greenhouse in winter in Raleigh. NC. This environment had relatively low temperature and light with poor air flow which reduced transpiration. As the weather warms up in the spring, the higher light and temperatures, combined with the air flow caused by the fans cooling the greenhouse, provide conditions in which transpiration rates are considerably higher. These conditions likely explain why we observe the Ca-deficiency phenotype more often in winter than in summer. For example an experiment conducted in May 2019 found symptoms in only about 60% of B73 plants with similar fertilizer treatment while in the winter months we generally expect to observe the phenotype in 100% of seedlings. However differing transpiration rates are unlikely to be the cause of the difference in sensitivity between lines, though, since B73 has been observed to have higher transpiration and lower water use efficiency than Mo17 (Blankenagel *et al.* 2018). It is possible that some of the QTL affecting Ca-deficiency that we identified in this study may be associated with regulating transpiration and water use efficiency of the plant.

### Competition between Ca^2+^ and other cations

The absorption of Ca**^2+^** into plant roots is inhibited by the presence of other cations such as NH_4_^+^, K^+^ Na^+^, H^+^ and Mg^2+^ (Bangerth 1979; Kirkby 1979; Wallace and Mueller 1980). While the exact mechanism of this inhibition has not been elucidated, it is likely because Ca**^2+^** uptake from the soil into the roots is mediated by non-selective cation channels for which other cations compete (Demidchik *et al.* 2002; Demidchik and Maathuis 2007). High NH_4_^+^ concentrations reduce Ca**^2+^** uptake in crops such as broccoli, tomato, pepper, (Claassen and Wilcox 2010) lettuce (Weil 2015) apple and potato (Kirkby 1979)

Similarly, in sorghum and maize, high levels of various cations, in particular NH_4_^+^, has been associated with Ca-deficiency (Wilcox *et al.* 1973; Kawaski and Moritsugu 1979; Murtadha *et al.* 2010). In our experiments we observed a similar phenomenon with increasing NH_4_/Ca ratios leading to Ca-deficiency symptoms (Figure 3, 4, Table S3).

Beyond the direct competitive interaction, the effect of NH_4_^+^ is likely detrimental to growth: high levels of NH_4_^+^ are well known to be toxic to plants in general (Britto and Kronzucker 2002), and the separate but interrelated effects of Ca-deficiency and ammonium toxicity have been previously discussed with regards to sudangrass (Bennett and Adams 1970). A high capacity for conversion and storage of NH_4_^+^ as amino acids is a proposed mechanism for NH_4_^+^ tolerance (Bittsánszky *et al.* 2015). Testcrosses of B73 and Mo17 (crossed to the same elite inbred) were found to differ in some aspects of nitrogen metabolism, especially in activity of glutamine synthetase, which is crucial for assimilation of NH_4_^+^ into amino acids (Silva *et al.* 2018).

Variation between maize lines with respect to Ca levels and Ca-deficiency phenotypes has been reported previously. A previous study (Clark and Brown 1974) identified variability among maize lines for Ca-deficiency symptoms, as well as to symptoms of deficiencies of other cations; Fe, Zn, Mg, Cu, Al and Mn. In every case, lines that displayed fewer symptoms had higher concentrations of the cation in their leaves. Variations in leaf Ca concentrations and a positive correlation between leaf-Ca and grain yield was reported among nine maize lines (Kovačević *et al.* 2017). Variation for kernel mineral concentration has been observed in the IBM population, and twenty-seven loci were found to be associated with the ionome variance (Baxter *et al.* 2013). However genetic loci associated specifically with variation in Ca-deficiency response have not been reported previously in maize, or, to our knowledge in any plant species.

### Analysis of QTL and candidate genes related to Ca-deficiency

To identify QTL associated with Ca-deficiency we used a RIL mapping population derived from a cross between two maize lines which showed significantly divergent Ca-deficiency symptoms; B73 in which the symptoms were very apparent, and Mo17 in which the symptoms were not observed. When grown in fertilizer-amended pots in the greenhouse, the 276 lines from this population showed substantial variability for the Ca-deficiency phenotype (Figure 5). The experiment was run for 14 days for the first replication but only seven days for the second. The reason for this was that the external (and therefore the internal greenhouse) temperature and light intensity, in the second experiment was higher than that in the first. Consequently, the plants grew faster and the Ca-deficiency phenotype appeared more quickly and did not change after seven days of cultivation. Nevertheless, the trait was highly heritable. We identified five QTL associated with variation in the appearance of the Ca-deficiency trait. At four of these QTL the allele for the appearance of the trait derived from B73 as expected, though in one case it derived from Mo17 (Table 2).

The two strongest QTL, located on chromosomes 2 and 6, were detected in both replications and in the overall data and were responsible for 9.9% and 8.3% of the observed variation respectively. The IBM mapping population provides relatively precise mapping accuracy due to the repeated intermating performed at the F_2_ stage during the construction of this population which led to a higher number of recombination events and the expansion of the genetic map (Lee *et al.* 2002; Balint-Kurti *et al.* 2007). From among 316 predicted genes at these QTL we identified 6 candidate genes with predicted functions associated with Ca. These are presented in Table S5. Zm00001d028582 on chromosome 1 encodes CPK5 (calmodulin-domain protein kinase 5) (Ye *et al.* 2009); Zm00001d006101 encodes a predicted calmodulin-binding and Zm00001d006066 encodes a predicted FK506-binding protein which is associated with a Ca release channel (Jayaraman *et al.* 1992; Noguchi *et al.* 1997). Zm00001d006136 encodes a predicted member of a Calcium-dependent phospholipid-binding protein family which is involved in plant defense signaling pathways (Lecourieux *et al.* 2006). Zm00001d038199 and Zm00001d038205 on chromosome 6 are MLO-like proteins, which carry a predicted calmodulin binding domain. It should be emphasized however that the causal genes may not have functions associated directly with Ca but may, for example, affect water movement in the plant or nitrogen assimilation or other processes we have not considered.

## Conclusions

In this study we have characterized a Ca-deficiency phenotype in maize and identified genetic factors associated with variation in the trait. Ca-deficiency is closely linked to transpiration and water movement in the plant and is strongly affected by high NH_4_^+^ levels. We have not determined the physiological basis of the greater tolerance to Ca-deficiency/high NH_4_^+^ levels in Mo17 compared to B73. Differences in transpiration may be one factor though Mo17 has been reported to have lower transpiration than B73, which argues against this being the main cause. It may be that Mo17 requires less Ca at the leaf tips or perhaps the mechanism is related to tolerance NH_4_^+^ toxicity, allowing tolerant lines to continue normal physiological function, including Ca uptake and transport. While Ca-deficiency is not a major limitation to maize cultivation, the further characterization of the genetics and physiology underlying variation in the Ca-deficiency phenotype may be of considerable interest since it is a relatively easily-scorable and highly heritable phenotype and might provide us an important window into factors affecting water relations and water transport in the xylem. These processes are important in modulating drought stress, an extremely important cause of yield loss in maize (Daryanto *et al.* 2016) and one that is likely to become more important with increasing climate volatility.
